# Case report: Abdominal mesothelioma in *Atelerix albiventris*

**DOI:** 10.3389/fvets.2024.1341815

**Published:** 2024-05-13

**Authors:** Ignacio Troncoso, John Brown, Carla Isla, Juan Manuel Lajara, Max Ebner, Karen Fehrmann-Cartes

**Affiliations:** ^1^Facultad de Medicina Veterinaria y Agronomía, Universidad de Las Américas, Concepción, Chile; ^2^Facultad de Recursos Naturales y Medicina Veterinaria, Universidad Santo Tomás, Sede Talca, Concepción, Chile; ^3^Servicio Patológico y Anestesiológico veterinario (SERPAVET), Concepción, Chile; ^4^Laboratorio Patológico Veterinario, San Isidro, Lima, Peru; ^5^Núcleo de Investigaciones Aplicadas en Ciencias Veterinarias y Agronómicas, Universidad de Las Américas, Concepción, Chile

**Keywords:** African pygmy hedgehog, neoplasms, fibrous mesothelioma, immunohistochemistry, calretinin

## Abstract

Hedgehogs, as exotic species, are more susceptible to various neoplastic conditions affecting diverse bodily systems, particularly the tegumentary, hemolymphatic, and digestive systems. Among these conditions, epithelial tumors are the most prevalent, followed by round cell tumors and mesenchymal tumors. A striking characteristic is the malignant nature of over 8% of these tumors, leading to a generally unfavorable prognosis. This study aims to present a unique case involving a 2.5 year-old male African pygmy hedgehog in Concepción, Biobío District, Chile, diagnosed with a mesenchymal neoplasia originating from mesothelial cells. The hedgehog presented to the veterinary clinic with acute abdominal pain, prompting ultrasound imaging, and comprehensive cytological, histopathological, and immunohistochemical analyses. During abdominal ultrasound, a mass was observed, and its cytological examination revealed the presence of malignant cells. The histopathological examination unveiled a diffuse mesothelial cell tissue interwoven with abundant fibrous tissue and small cysts containing serous fluid, all enveloped by flattened or cuboidal cells of mesothelial origin. Immunohistochemistry further confirmed the diagnosis, demonstrating positive immunostaining for calretinin and mesothelin markers, corroborating the diagnosis of fibrous malignant peritoneal mesothelioma. This case highlights the complexity of neoplastic conditions in hedgehogs and emphasizes the importance of multimodal diagnostic approaches for accurate identification and understanding of these rare diseases.

## 1 Introduction

African pygmy hedgehogs (*Atelerix albiventris*), also known as four-toed hedgehogs, are small, spiny insectivores that have become increasingly popular as domestic animals in many countries in the Americas, Asia, and Europe (1). To ensure the wellbeing of these small, spiny insectivores, owners are advised to schedule veterinary check-ups every 6 months. This precaution is particularly crucial as African pygmy hedgehogs often conceal signs of pathology until the conditions reach an advanced stage, resulting in significant organ damage (2). The increasing prevalence of hedgehogs as companion animals has yielded valuable insights into their health and diseases. Notable conditions observed include periodontal disease, traumatic exophthalmia, bronchopneumonia, dilated cardiomyopathy, bacterial gastroenteritis, renal failure, endometrial polyps, limb constriction by hairs or threads, wobbly hedgehog syndrome, *Caparinia tripilis* acariasis, pyoderma, and neoplasms (3).

Neoplastic processes have been identified as one of the leading causes of mortality in captive hedgehogs, accounting for 25% of clinical problems (4–6). The average age of hedgehogs with neoplasms is approximately between 2.5 and 3 years (6), with a life expectancy of 5 years and exceptionally up to 8 years (7). In many studies, epithelial neoplasms are the most common, followed by round cell tumors and mesenchymal cell tumors (5–7). In addition to the mentioned neoplasms, there are other less frequent neoplasms such as endometrial stromal sarcomas, leiomyoma, adenoleiomyoma, adenocarcinoma, lymphoma, oral squamous cell carcinoma, schwannoma or neurofibrosarcoma, plasma cell tumors, hemangiosarcoma, fibrosarcoma, osteosarcoma, undifferentiated or poorly differentiated sarcomas, mammary gland tumors, mast cell tumors, sebaceous carcinoma, and lipoma (8). Additionally, it is important to note that the rate of malignancy is high (up to 85%), which often results in death (5, 6).

Consequently, a deeper understanding of neoplastic processes, including their incidence, pathophysiological behavior, and diagnostic approaches, is essential for informed treatment planning and accurate prognosis assessment. To contribute to the expansion of knowledge and provide a better understanding of neoplastic processes affecting hedgehogs, this study presents a report on specific type of neoplasm called “fibrous abdominal mesothelioma.” Mesothelioma is a type of cancer formed in the layer of cells that cover internal organs (7). This contribution aims to enhance the collective understanding of such conditions within the scientific community.

## 2 Case

### 2.1 Patient clinical examination

A 2.5-year-old male African hedgehog species was brought in consultation at a veterinary clinic in Concepción, Biobío District, Chile, on 21 January, 2022. The main clinical sign observed was intense pain and acute abdomen palpation of the area, although no mass was detected. Blood tests, biochemical profile, and abdominal ultrasound were requested as complementary examinations, which were performed in a veterinary clinical laboratory located in Concepción.

### 2.2 Abdominal ultrasound

The patient underwent an ultrasound examination, the day after the consultation, and the results showed the following: slight dilation of right renal pelvis due to an anechoic content (0.14 cm thick), with accompanying dilation of the ipsilateral ureter; a spleen of heterogeneous appearance, with irregular contours; a skull mass ventral to the bladder silhouette, heterogeneous in appearance, hypoechoic, which measures 1.22 cm in diameter, and adjacent to a jejunal intestinal loop; and a small amount of free anechoic content in the abdominal cavity. The ltrasound analysis was performed with SONOSITE^®^ m Turbo equipment and a transducer HFL50 de 15 MHz.

### 2.3 Additional test and cytology

Hematology showed lymphocytosis, monocytosis, and eosinophilia with hypersegmented neutrophils, mild anisocytosis, and hypochromic, poikilocytosis with a predominance of acanthocytes and the presence of platelet aggregates. Blood biochemistry showed an increase in the level of cholesterol (Supplementary Table S1).

After visualization of the mass through ultrasound imaging, a cytological analysis was performed. The collection of tissue samples was carried out by fine needle aspiration puncture (FNAC) using a 21′–25′ G needle attached to a 3–5 mL syringe (Sigma-Aldrich). Negative pressure was applied several times while redirecting the needle in several parts to ensure homogenous sampling. The sample obtained was spread on a slide by placing another slide on top following Perez (2019) (9) for intra-abdominal masses, dry fixation (10), and Diff-Quik staining (11).

The cytological evaluation of the attached mass showed moderate cellularity in the smear obtained by FNAC, with degenerated neutrophils, erythrocytes, and eosinophils. A few oval-shaped cells with moderate pleomorphism were observed, meeting nuclear malignancy criteria, such as anisokaryosis, nucleoli, and a nucleus-to-cytoplasm ratio of 1:1 as well as cytoplasmic malignancy criteria, such basophilia (Supplementary Figure S1).

### 2.4 Tumoral mass removal

**Figure 1:**
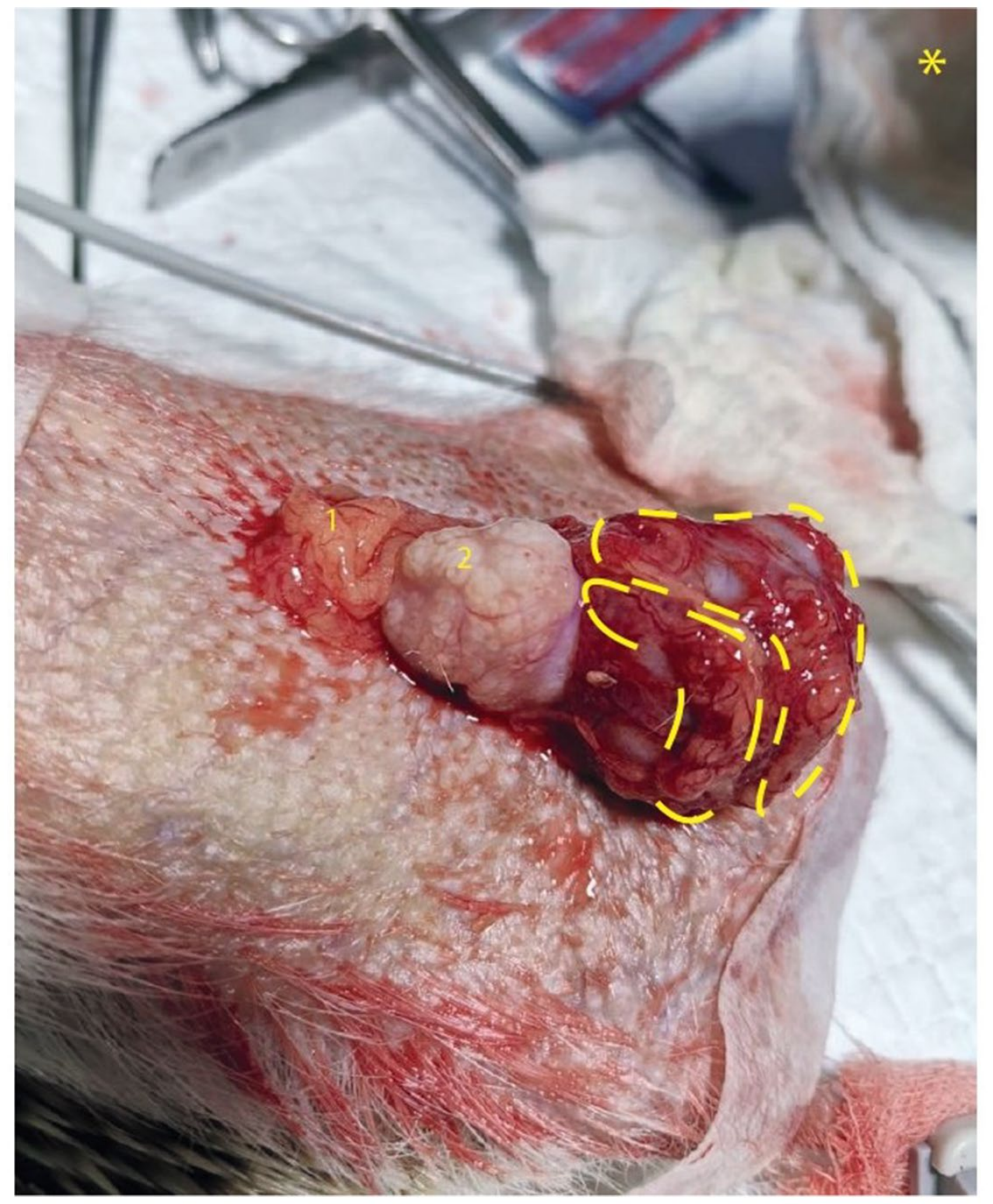
Peritoneal tumor in Hedgehog. **(A)** Mass of mesenchymal origin adhered to the bladder (marks in yellow show two lobes of tumor mass). 1 Jejunum, 2 Seminal vesicle, * shows caudal orientation in image.

Approximately 2 weeks later, a surgery was scheduled to remove the observed mass. Prior to surgery, a second ultrasound analysis was performed to confirm previous observations. Before the operation, premedication was administered with Ketamine at a dose of 25 mg/kg and Midazolam at 1 mg/kg. For anesthetic induction, Isoflurane is utilized at 5% and for maintenance, it is used at 2%. Meloxicam at a dosage of 0.2 mg/kg and Tramadol of 2 mg/kg were administered for analgesia. The surgery was performed through an abdominal midline approach, and the abdominal tumor mass was surgically removed. The tumor was located in the peritoneal cavity and adhered ventral to the bladder, caudal to seminal vesicles and cranial to prostate. From visual and morphological points of view, the presence of two oval, whitish, granular, well-defined, and solid lobes was observed; one was larger than the other (measuring 2.5 cm and 1.5 cm, respectively) (Figure 1).

### 2.5 Histopathology and immunohistochemistry

The sample was obtained through excisional biopsy, taking a fragment of neoplastic tissue from the mass removed from the patient. The tissues were initially immersed in a 10% formaldehyde fixative solution immediately after the removal from the patient. At the end of fixation, the fixative was removed from the samples using bidistilled water wash. After washing, the samples were dehydrated and infiltrated with a solvent suitable for paraffin infiltration (12). The paraffin-tissue block was cut between 4 and 10 μm slices using a microtome. Finally, the sample was stained using hematoxylin–eosin (H-E) and examined under an optical microscope (Leica DM 2000). To confirm the diagnosis based on observations made during the tissue biopsy histopathology, we carried out immunostaining using calretinin (mesothelioma sensitive marker (13)), mesothelin (tumor differentiation antigen overexpressed in neoplasms (14)), and cytokeratin 5/6 (CK 5/6), which are proteins with expression in stratified epithelial layers, mesothelial cells, and proliferating squamous epithelium (15). The tissue sections were then incubated with antibodies against calretinin (mouse, clone H5, 1: 100, Diagnostic Biosystem), mesothelin (human, clone 5B2, 1: 50, Thermo Fisher), and cytokeratin 5/6 (mouse, clone D516 B4, 1:100, Diagnostic Biosystem). Following this, the Mouse/Rabbit PolyDetector DAB HRP Brown detection system (Bio SB, Germany) was used according to manufacturer’s instruction. Finally, the slides were counterstained with hematoxylin.

In the negative controls, the primary antibody was replaced with non-immune serum. Positive and negative tissue controls included human tissue. Normal hedgehog tissue positive for calretinin, mesothelin, and cytokeratin 5/6 was not available.

**Figure 2:**
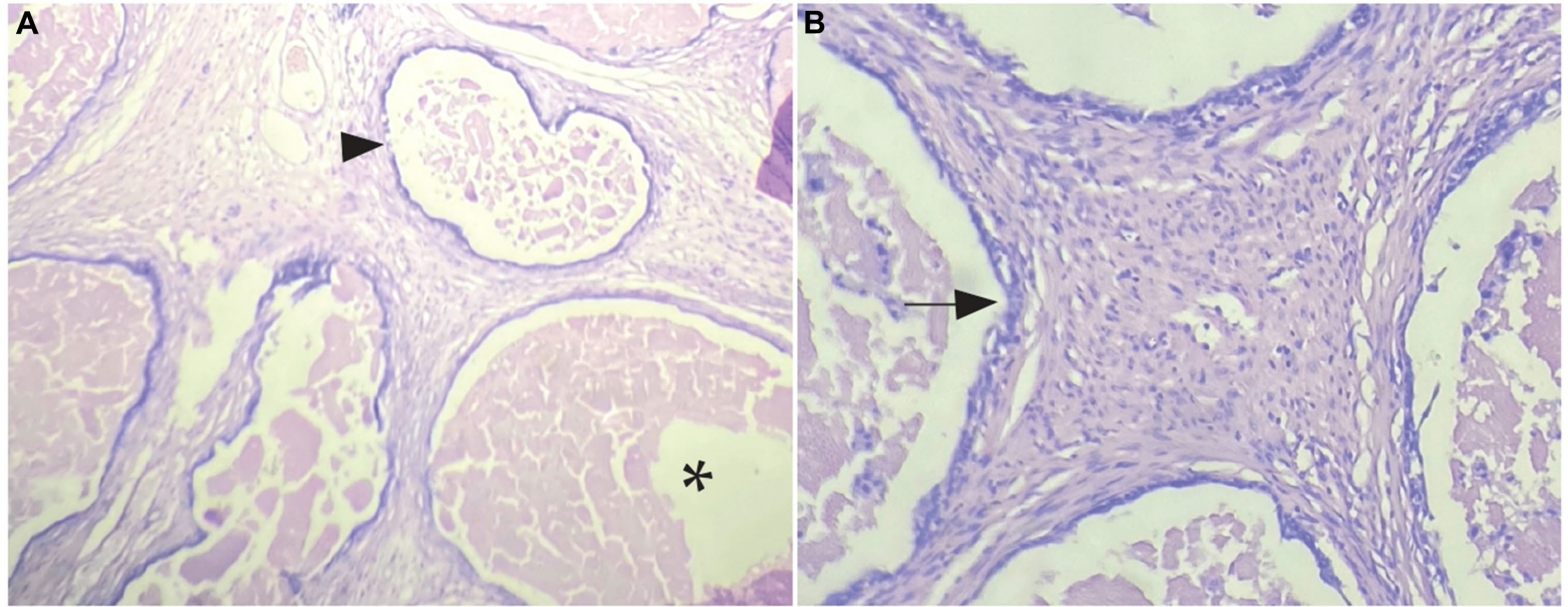
Histopathological sample with H-E staining. Mesothelial cells arranged in solid masses. **(A)** Arrow heads show small cysts covered by neoplastic mesothelial cells, asterisk marks interior of cysts with fluid and detached cells inside (4× magnification). **(B)** Arrow marks mesothelial cells bordering the cyst (10× magnification).

The histopathological evaluation, using H-E staining, showed a diffused non-encapsulated tissue composed of a few mesothelial cells arranged in solid cysts masses along with large amounts of non-neoplastic fibrous tissue on the exterior. Some areas presented small cysts filled with serous fluid and were also observed to be covered by flattened or cuboidal mesothelial cells (Figures 2A,B).

**Figure 3:**
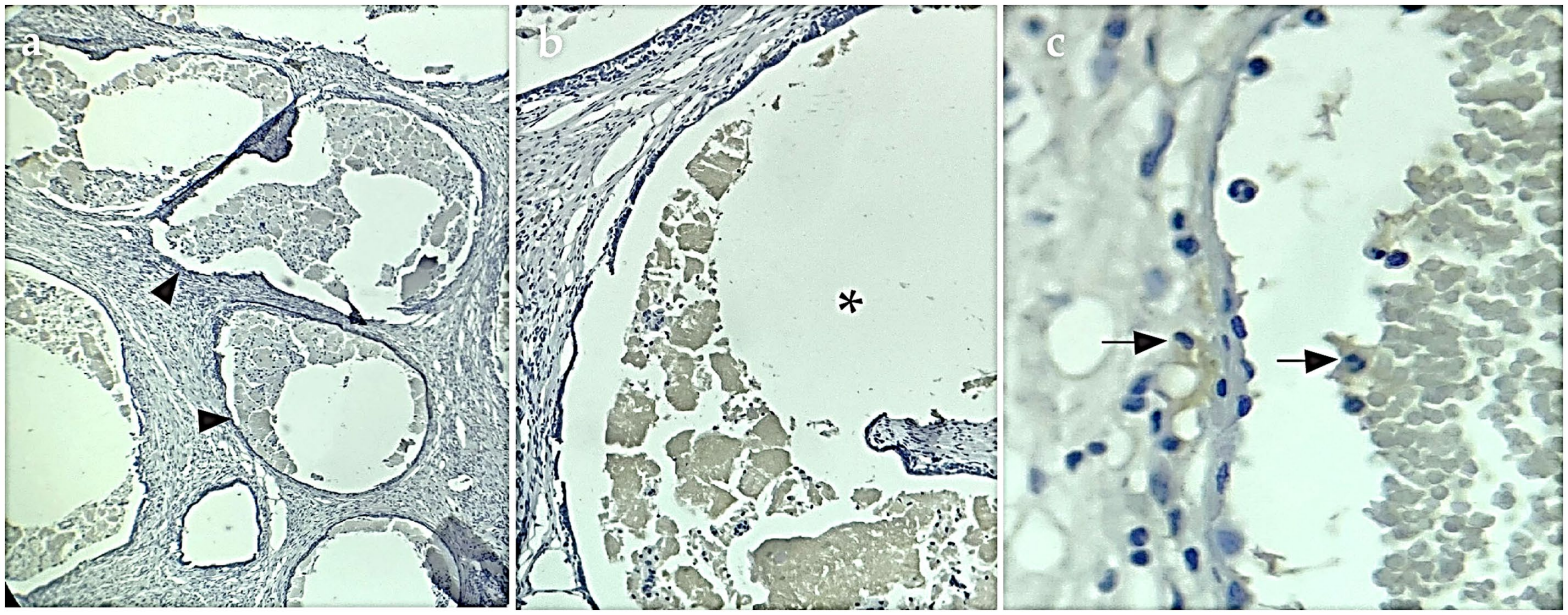
Calretinin-positive IHC sample. **(A)** IHQ with 4× magnification, arrowheads show cysts surrounded by cells positive for the calretinin marker. **(B)** Asterisk shows interior of cysts with fluid and detached cells inside, 10× magnification. **(C)** 40× magnification arrows mark cells labeled with IHQ brown staining.

**Figure 4:**
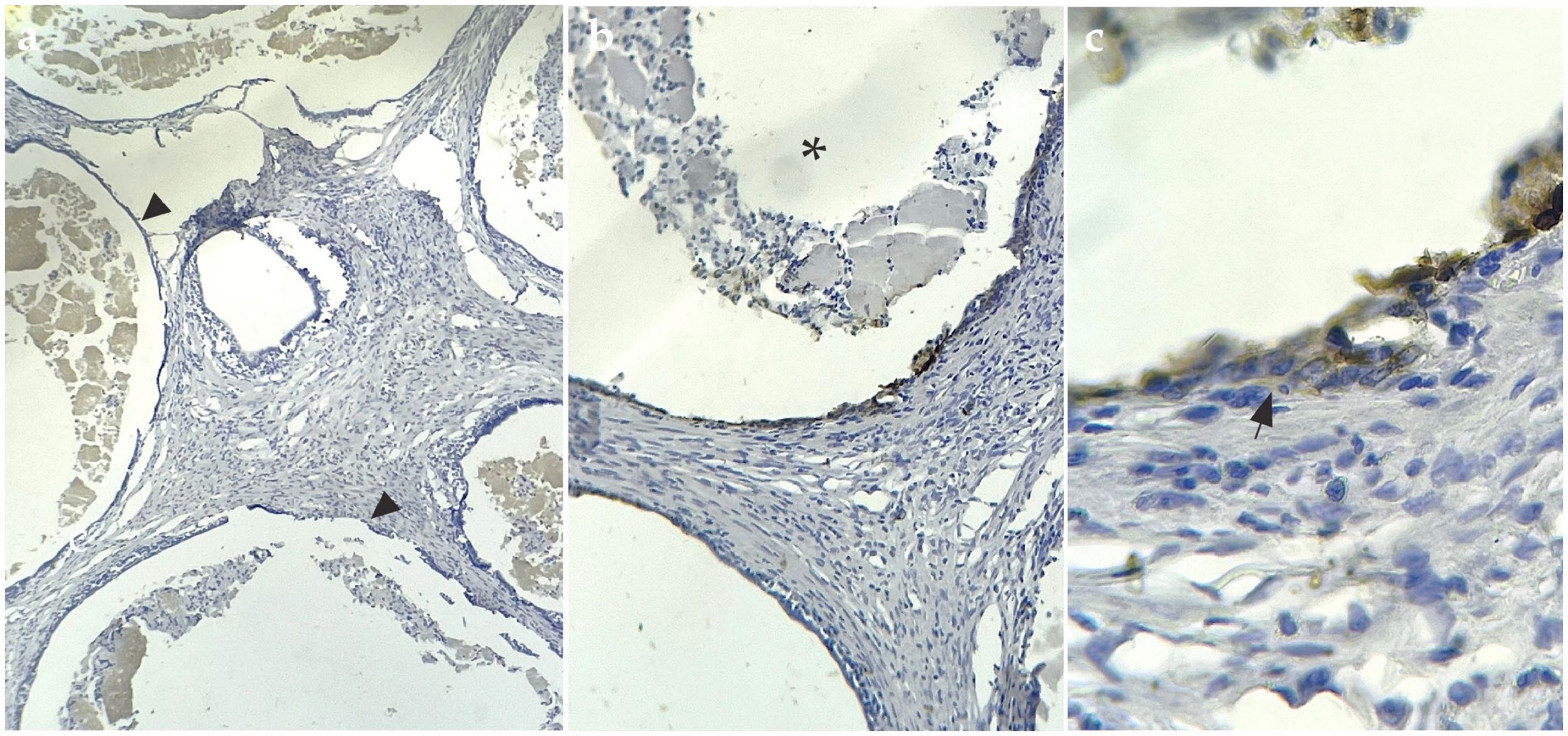
Mesothelin-positive IHC sample. **(A)** IHQ with 4× magnification, arrowheads show cysts surrounded by cells positive for the mesothelin marker. **(B)** Asterisk shows interior of cysts with fluid and detached cells inside, 10× magnification. **(C)** 40× magnification arrows mark cells labeled with IHQ brown staining.

IHC staining showed evident cytoplasmatic positive immunostaining for the marker calretinin (Figure 3) and mesothelin (Figure 4), but showed no positivity for CK5/6. This confirmed the final diagnosis of fibrous mesothelioma (16).

## 3 Discussion

Initially, in this clinical case, because the mass was found in the abdomen, gastrointestinal lymphoma was suspected. This type of tumor of the hemolymphatic system is the second most common in land hedgehogs, corresponding to 11% of the neoplasms in this species. Since it is a round cell tumor, it has a strong diagnostic approach in cytology, which is why it was decided to take this type of sample in the first instance, finding no compatibility of findings (7). The cytological evaluation revealed the presence of moderate cellularity, with degenerated neutrophils. In addition, cells exhibited oval morphology of moderate pleomorphism, nuclei were ovoid in shape, and there was an increased nucleus: cytoplasm ratio. In mesotheliomas, it is common to find neoplastic cells that can vary from exclusively epithelial types to mixed forms, where characteristic pointed cells are also found (17).

The histological and morphological characteristics of the tumor in this study are similar to those of canine species mesothelioma. Mesotheliomas are rare neoplasms described in different species such as bovines, horses, canines, felines, and humans (18). The etiology of mesothelioma in hedgehogs is still unknown. Mesotheliomas have been associated with chronic asbestos inhalation in humans and canines (19, 20). There are few cases with the presence of asbestos bodies in dogs (21). Several mechanisms through which asbestos leads to benign and malignant diseases have been reported. However, there are currently no effective means to determine which patients exposed to asbestos will develop malignant neoplasms and which will not (22–25).

The microscopic histopathological results are similar to those previously described for canine mesothelioma, where a proliferation of neoplastic mesothelial cells has been observed. These cells form solid areas or cover cystic spaces and tubular structures, presenting a mucinous matrix pattern (26). The neoplastic cells are classically cuboidal with scant to moderate amounts of eosinophilic cytoplasm (27), supporting the diagnosis of mesothelioma. Generally, these tumors show marked anisocytosis and anisokaryosis, pleomorphism, bi-, and multinucleation, with mitosis occurring more frequently (28).

The result from the IHC staining demonstrated a high specificity for both normal and neoplastic mesothelium cells which are similar to those described using the calretinin marker in humans with this type of tumor (29). Additionally, the sections of the excised tissue were positive for mesothelin, a marker that participates in cell adhesion and cell–cell recognition, which is expressed in normal tissues and is overexpressed in different tumors such as malignant mesothelioma in humans (30). The tumor cells were negative in the for cytokeratins 5/6 (CK5/6) in the immunohistochemical study. These immunohistochemical markers of epithelioid mesothelioma are considered as valuable by authors (31).

Immunohistochemestry (IHC) markers are not validated for the species of this study; however, according to the prospectus, they present reactivity in other species, with reactivity to 80 a 100% (32), its demonstrated when polyclonal antibodies against human recombinant calretinin are used, calretinin is 80–100% sensitive for malignant mesothelioma (33, 34), with matches (35), when calretinin are positive in 95% of epithelioid mesotheliomas; staining is often strong and diffuse and must be both nuclear and cytoplasmic. While to mesothelin showed sensitivity and specificity of 0.73 (CI, 0.68–0.77) and 0.90 (CI, 0.84–0.93), respectively (36).

In a 2007 study, researchers confirmed the value of cytokeratin 5/6 as a marker of mesothelioma in effusion samples. Effusion, the buildup of excess fluid, is a common symptom of pleural and peritoneal mesothelioma, and this is similar as in this study (37). This marker is not effective for all mesothelioma cell types, it is weak or negative for fibrous mesothelioma, which is the least common and most difficult to treat asbestos-related cancer cell type in domestic animals (38). However, this marker showed reactivity to 51% of peritoneal mesothelioma cells (35). Mesothelioma has three distinct histological patterns, namely epithelioid, fibrous (sarcomatoid), and mixed cell (biphasic). These tumors may present foci of chondroplasia and osteoid formation or mineralized bone, which were not present in this case. The fibrous variant of malignant mesothelioma somewhat resembles fibrosarcoma, while the biphasic variant contains areas of carcinomatous and sarcomatoid features (39). Each type can be diffuse or localized. In the first group, the epithelial and mixed types predominate, while among the localized forms, the fibrous type is more common (40). Additional subtypes reported in canine species include deciduoid peritoneal mesothelioma, cardiac mesothelioma with granular cell morphology, lipid-rich pleural mesothelioma, and cystic peritoneal mesothelioma (41). Macroscopically, mesotheliomas appear primarily as fragile, pedunculated nodules with villous projections on the serosal surface (42). In contrast, sclerosing (mesenchymal) mesotheliomas are large, firm, multinodular, irregularly shaped masses with smooth surfaces (41), which tend to spread and thicken through fibrous adhesions on the serous surfaces of the abdomen. Affected animals show severe decline and are generally diagnosed clinically as having generalized peritonitis (42). In this clinical case, there were several points of interest: on the one hand, there is difficulty in diagnosing this type of neoplasia that is not described previously for this species. There have been no studies describing ultrasound results in hedgehogs, and it was difficult to distinguish the neoplastic nodules due to the hydronephrosis present and the accumulation of intra-abdominal fluid, which required a second examination to confirm the presence of a mass. Furthermore, the cytological characteristics of reactive mesenchymal cells due to inflammatory processes are very similar to those of neoplastic mesenchymal cells (29, 40). On the other hand, the biopsy after the removal of the mass was the method that allowed us to analyze the morphological findings, cyto- and anatomopathological findings and the immunohistochemical reactivity, confirming the suspicion of mesothelioma. Finally, the morphological characteristics described in the results led to the final diagnosis of localized malignant fibrous peritoneal mesothelioma, demonstrating compliance with at least three nuclear criteria for cellular malignancy (43).

Approximately 2 weeks after the tumor removal surgery, the hedgehog was monitored, showing clinical improvements, without signs of pain and with recovery of appetite. However, 1 month after this check-up, the owners reported the death of the hedgehog, following an acute deterioration over 2 days.

## 4 Conclusion

In conclusion, the microscopic lesions identified exhibit characteristics indicative of neoplastic transformations arising from mesenchymal cells with a mesothelial origin within the abdominal serosa, specifically of a fibrous nature. This study serves to highlight the viability of fibrous mesothelioma as a plausible diagnosis for abdominal neoplasia in terrestrial hedgehogs.

## Data availability statement

The original contributions presented in the study are included in the article/Supplementary material, further inquiries can be directed to the corresponding author.

## Ethics statement

The animal studies were approved by Comité Ético Científico de UDLA. The studies were conducted in accordance with the local legislation and institutional requirements. Written informed consent was obtained from the owners for the participation of their animals in this study.

## Author contributions

IT: Formal analysis, Investigation, Methodology, Supervision, Validation, Visualization, Writing – original draft, Writing – review & editing, Software. JB: Data curation, Formal analysis, Supervision, Validation, Writing – original draft. CI: Conceptualization, Data curation, Investigation, Methodology, Writing – original draft. JL: Formal analysis, Investigation, Methodology, Software, Writing – original draft. ME: Conceptualization, Data curation, Methodology, Visualization, Writing – original draft. KF-C: Data curation, Investigation, Methodology, Visualization, Writing – original draft.
